# Reappraising the role of cardiac magnetic resonance in heart failure with preserved ejection fraction

**DOI:** 10.1016/j.ihj.2026.04.009

**Published:** 2026-04-28

**Authors:** Barbara Segulin, Nikolaos Miaris

**Affiliations:** Harefield Hospital, Royal Brompton and Harefield Hospitals, Guy's and St Thomas' NHS Foundation Trust, Harefield, UK

To the Editor,

One of the most frequent indications for cardiac magnetic resonance (CMR) in clinical practice is the investigation of the aetiology of left ventricular systolic dysfunction, for which it represents the gold standard for accurate volumetric assessment and comprehensive tissue characterisation. Accordingly, its role in the evaluation of ischaemic heart disease and a broad spectrum of cardiomyopathies is well established.

In their recently published study in the journal, Mondal et al showed that, in a cohort of patients with heart failure with preserved ejection fraction (HFpEF), those with no late gadolinium enhancement (LGE) had higher myocardial T1 values than controls.^1^ Importantly, the study highlighted the prognostic relevance of myocardial T1 values, as HFpEF patients who experienced adverse events (heart failure hospitalisation, stroke, or all-cause mortality) had higher values than those without events. Myocardial T1 values, as a surrogate of interstitial myocardial fibrosis not captured by LGE imaging, may therefore provide prognostic information. Patients with HFpEF and controls did not have significantly different T2 values.

Although not explicitly stated in the exclusion criteria, the authors noted that patients with coronary artery disease, hypertrophic cardiomyopathy, amyloidosis, and restrictive cardiomyopathy were not included, which strengthens the clinical relevance of the findings. This study adds to the growing body of evidence supporting the role of CMR in the evaluation and risk stratification of HFpEF. A meta-analysis by Golukhova et al showed that higher T1-derived ECV in HFpEF patients, but not native T1 values per se, was associated with worse prognosis (risk of death and/or hospitalisation with heart failure).^2^ Additionally, Romano et al demonstrated the prognostic value of CMR global longitudinal strain in patients with preserved ejection fraction undergoing CMR.^3^ Although the presence of LGE did not affect prognosis in the current study by Mondal et al, the amount of myocardial fibrosis, as assessed by LGE quantification, has shown prognostic value in the study by Kato et al in a cohort of HFpEF patients in Japan.^4^

To conclude, despite potential heterogeneity in study populations and technical variations, the current evidence expands the role of CMR in HFpEF for risk stratification, beyond phenotyping, with the aim of intensifying treatment and management of associated conditions and comorbidities in identified high-risk individuals.

## Declaration of competing interest

The authors declare that they have no known competing financial interests or personal relationships that could have appeared to influence the work reported in this paper.
